# Evaluation of Locally Established Reference Intervals for Hematology and Biochemistry Parameters in Western Kenya

**DOI:** 10.1371/journal.pone.0123140

**Published:** 2015-04-13

**Authors:** Collins Odhiambo, Boaz Oyaro, Richard Odipo, Fredrick Otieno, George Alemnji, John Williamson, Clement Zeh

**Affiliations:** 1 Kenya Medical Research Institute, Kisumu, Kenya; 2 U.S. Centers for Disease Control and Prevention (CDC), Bridgetown, Barbados; 3 U.S. Centers for Disease Control and Prevention (CDC), Kisumu, Kenya; University of Cape Town, SOUTH AFRICA

## Abstract

**Background:**

Important differences have been demonstrated in laboratory parameters from healthy persons in different geographical regions and populations, mostly driven by a combination of genetic, demographic, nutritional, and environmental factors. Despite this, European and North American derived laboratory reference intervals are used in African countries for patient management, clinical trial eligibility, and toxicity determination; which can result in misclassification of healthy persons as having laboratory abnormalities.

**Methods:**

An observational prospective cohort study known as the Kisumu Incidence Cohort Study (KICoS) was conducted to estimate the incidence of HIV seroconversion and identify determinants of successful recruitment and retention in preparation for an HIV vaccine/prevention trial among young adults and adolescents in western Kenya. Laboratory values generated from the KICoS were compared to published region-specific reference intervals and the 2004 NIH DAIDS toxicity tables used for the trial.

**Results:**

About 1106 participants were screened for the KICoS between January 2007 and June 2010. Nine hundred and fifty-three participants aged 16 to 34 years, HIV-seronegative, clinically healthy, and non-pregnant were selected for this analysis. Median and 95% reference intervals were calculated for hematological and biochemistry parameters. When compared with both published region-specific reference values and the 2004 NIH DAIDS toxicity table, it was shown that the use of locally established reference intervals would have resulted in fewer participants classified as having abnormal hematological or biochemistry values compared to US derived reference intervals from DAIDS (10% classified as abnormal by local parameters vs. >40% by US DAIDS). Blood urea nitrogen was most often out of range if US based intervals were used: <10% abnormal by local intervals compared to >83% by US based reference intervals.

**Conclusion:**

Differences in reference intervals for hematological and biochemical parameters between western and African populations highlight importance of developing local reference intervals for clinical care and trials in Africa.

## Introduction

The burden of diseases such as HIV/AIDS, tuberculosis, and malaria is heaviest in sub-Saharan Africa compared to the rest of the world [1, 2]. For example, sub-Saharan Africa has the highest prevalence and incidence of HIV infection globally. As such, a major of many recent HIV prevention, care and treatment initiatives are being conducted within the region [3, 4]. including most phase I ⁄ IIB HIV-1 vaccine trials [5].

With increasing clinical trials in sub-Saharan Africa to combat these diseases, there is a need for accurate clinical laboratory reference intervals for appropriate participant screening, disease progression monitoring and evaluation of possible clinical trial-associated toxicity and adverse events [6]. Reference intervals are important for guiding patient treatment and management as well as identifying abnormal hematologic values [7]. For example, the complete blood count and CD4 determination are important laboratory tests in HIV-endemic regions [8]. The level of hemoglobin concentration has utility as a prognostic indicator while CD4 is used to make decisions regarding initiation of antiretroviral drugs and to monitor disease progression. These tests require accurate reference intervals for correct interpretation of laboratory results. However, currently used reference intervals in many countries in sub-Saharan Africa are derived from populations in Europe and North America [6, 9]. Since hematologic parameters are affected not only by individual factors such as age, sex and lifestyle, but also by population and ecological factors such as ethnic background, climate, exposure to pathogens and altitude, they vary not only between individuals but also between populations [10]. Thus, there is not a universal definition of ‘normal’ hence it is important to define reference intervals that are suited to the particular population of interest [10]. A few studies conducted in Africa over the last decade have highlighted differences in hematologic parameters between the local population and Caucasian populations in Europe and North America [11–15]. More recently, a study highlighted differences in hematological and biochemistry values between adolescent and young adult males [15]. Despite these recorded inter-population differences in reference values for different geographical regions, few data exist in Africa to provide locally-derived values [7, 12, 13, 16].

Despite these recorded differences, the Division of AIDS (DAIDS) National Institute of Health toxicity tables [17], are still used for grading the severity of adult and pediatric adverse events, whether or not they are considered to be related to the study intervention. This leads to unnecessary exclusion of would be participants misclassified as having abnormal hematologic parameters thereby escalating operational costs especially in phase I safety trials where there may not be a control group [18–20]. This may also lead to improper patient management through misclassification of adverse events. Due to these differences, there is a need to develop and test locally-derived age specific reference intervals within African populations.

While it is desirable to generate reference intervals for different populations, the procedure remains a challenge due to the prohibitive cost involved in performing these studies and the limitation in identifying suitable healthy reference individual. Thus, the recommendation by the Clinical and Laboratory Standards Institute (CLSI) that all diagnostic laboratories must determine and maintain their own reference interval for each laboratory parameter is impractical. CLSI further recommends that if it is not possible to establish the detailed reference studies, then validation of published reference intervals can be performed using own methodology for the population served by the laboratory. Zeh et al have recently established reference intervals for use in western Kenya [15]. These intervals were generated from a study conducted on 13–34 year old, clinically healthy, HIV-seronegative, non-pregnant residents of western Kenya. Because the established reference intervals were from a population in Siaya County in western Kenya, our aim was to validate these established reference intervals for use in Kisumu County of western Kenya. We also retrospectively determined the proportion of participants in an observational prospective cohort study known as the Kisumu Incidence Cohort Study (KICoS), who would be misclassified as having abnormal hematological parameters using the established reference intervals and compared our findings to those obtained using the 2004 NIH DAIDS toxicity tables.

## Materials and Methods

### Study population

This analysis utilized 953 samples obtained from 1106 participants screened in the KICoS conducted between January 2007 and June 2010 at the KEMRI/CDC Clinical Research Center (CRC) within New Nyanza Provincial General Hospital, Kisumu. The laboratory where the study was conducted is accredited by the South African National Accreditation System [21].

KiCoS was an observational prospective cohort study designed to estimate the incidence of HIV seroconversion and to identify determinants of successful recruitment and retention in preparation for an HIV vaccine or prevention trial among young adults and adolescents in Kisumu, western Kenya. Healthy adolescent (16–17 years) and young adult (18–34 years) residents of Kisumu who reported having sexual intercourse at least once in the past three months were eligible for the study. The study was conducted in the catchment area of Kisumu, a city of approximate population of 578,865 as projected by central bureau of statistics by 2006 in western Kenya [22]. All participants underwent screening for HIV-1 and HSV-2 among other sexually transmitted infections. Signs and symptoms were collected both in a self administered Audio Computer Assisted Self Interview (ACASI) (for STI symptoms) and a clinician administered Computer Assisted Personal Interview (CAPI) for all other symptoms. Blood samples were collected for complete blood count, HIV and HSV-2 testing with laboratory results.

### Ethical approval

Ethical approval for the study was obtained from KEMRI and CDC ethics review committee/institutional review board. Written informed consent was obtained from each participant prior to study initiation. Minors (<18 years of age) were classified as “mature” or “non-mature” using legal definitions [23]. Mature minors could consent to study participation as they would for HIV counseling and testing in Kenya. Non-mature minors went through a two-step written consent process involving consent from the parent or guardian followed by written individual assent from the minor.

### Blood collection and HIV serology

Whole blood was collected in EDTA vacutainer tubes (Becton Dickinson, Franklin Lakes, NJ) and transported to the KEMRI/CDC HIV-research laboratory for processing and analysis within six hours of specimen collection. HIV status was determined from whole blood using HIV rapid test kits as follows: Determine (Abbot Laboratories, Tokyo, Japan), and Unigold (Trinity Biotech Plc, Bray, Ireland), with Bioline (Standard Diagnostics Inc., Korea) as a tie breaker.

### Pregnancy testing

A urine pregnancy test was administered to all females who were not visibly pregnant, using First Sign HCG One Step (UNIMED International, Inc., South San Francisco, CA, USA).

### Hematological analysis

Absolute white blood cell counts and percentages for leukocytes (WBC) with differentials (neutrophils, lymphocytes, monocytes, eosinophils, and basophils), erythrocytes (RBC) with parameters (hemoglobin (Hb), hematocrit (Hct), MCV, and MCH), and platelet counts were determined from whole blood using a Coulter ACT 5Diff CP analyzer (Beckman Coulter, France). This was performed within 24 hours of sample collection as recommended by the manufacturer.

### Biochemistry analysis

Clinical chemistries were analyzed from serum obtained from serum separation tubes (Becton Dickinson, Franklin Lakes, NJ). Samples were analyzed for alanine aminotransferase (ALT), creatinine (Cr), and blood urea nitrogen (BUN) using the Cobas Integra 400 plus biochemistry analyzer (Roche, Germany) per the manufacturer’s instructions.

### Quality Control

Quality control protocols included running known standards each day before testing samples. In addition, the laboratory is enrolled in external quality assurance testing programs with the College of American Pathologists (lymphocyte immunophenotyping, hematology, and clinical chemistry) and the United Kingdom National External Quality Assurance Service (lymphocyte immunophenotyping). The laboratory has satisfactory performance in UK NEQAS (Lymphocyte Immunophenotyping) and CAP Clinical Chemistry as well as CAP Hematology over the past three years.

### Statistical analysis

Data were collected on optical character recognition (OCR) enabled forms and entered with scanners. Cross-checking and data cleaning was performed regularly We followed the guidelines of the Clinical Laboratory Standards Institute (CLSI, Wayne, PA, USA) for reference interval determination [10]. While these guidelines are meant for establishing new reference intervals, the basic principles also apply to validation of reference intervals [8]. The median and the 2.5 and 97.5 percentiles were calculated for each hematological parameter. Study participants were partitioned into two age groups: those 17 and younger (adolescents) and those 18 and older (young adults) and analyzed using SAS v9.1 (Cary, NC, USA). The Wilcoxon test was used to compare hematological parameters between the two age groups separately for males and females and to compare males and females separately by age group. A two-sided P value of ≤0.05 was considered significant.

We compared our data against reference intervals from the established reference intervals for western Kenya (Table 1), the Massachusetts General Hospital (MGH), USA reference intervals and the U.S. NIH Division of AIDS (DAIDS) toxicity tables, to determine the number (and percentage) of study participants who had values outside the established reference intervals (2.5 to 97.5 percentiles) or who had any adverse events as graded by the DAIDS criteria.

**Table 1:** Hematological, immunologic and biochemistry reference intervals (median and 95^th^-percentile) stratified by age and gender from a 13–34 years old cohort in rural western Kenya (2003–2005) [15].

	Age 13–17 years		Age 18–34 years	
Parameter	Male	Female	Male	Female
**RBC (10** ^**6**^ **Cells/μl)**	4.9 (4.1–5.8)	4.7 (3.3–5.4)	5.3 (4.3–6.5)	4.5 (3.4–5.7)
**Hb (g/dL)**	13.1(10.6–15.6)	12.2 (8.1–14.2)	14.2 (11.4–16.9)	12.1 (8.0–14.2)
**HCT (%)**	38.8 (29.3–48.1)	35.6 (24.8–43.1)	41.7 (32.6–51.5)	35.8 (23.2–44.3)
**MCV (fL)**	79 (62–92)	78 (57–91)	80 (55–98)	79 (60–94)
**PLT (10** ^**3**^ **cells/μl)**	224 (103–386)	233 (134–439)	201 (102–307)	220 (88–439)
**WBC(10** ^**3**^ **cells/μl)**	5.6 (3.3–8.3)	5.2 (3.9–10.2)	5.3 (2.5–7.4)	5.6 (3.3–9.7)
**Ne (10** ^**3**^ **cells/μl)**	1.9 (0.8–5.0)	2.0 (1.1–3.1)	2.0 (0.8–3.9)	2.3 (1.3–3.8)
**Ly (10** ^**3**^ **cells/μl)**	2.2 (1.0–4.2)	2.2 (1.1–3.1)	2.2 (1.0–3.5)	2.2 (1.3–3.8)
**Mo (10** ^**3**^ **cells/μl)**	0.5 (0.2–0.7)	0.4 (0.2–0.7)	0.5 (0.2–0.9)	0.5 (0.3–0.8)
**Eo (10** ^**3**^ **cells/μl)**	0.4 (0.1–1.8)	0.4 (0.1–2.2)	0.5 (0.1–1.7)	0.4 (0.1–1.3)
**Ba (10** ^**3**^ **cells/μl)**	0.04 (0.02–0.30)	0.04 (0–0.10)	0.04 (0.01–0.19)	0.04 (0–0.20)
**CD4 (10** ^**3**^ **cells/mm** ^**3**^ **)**	874 (367–1571)	934 (465–1553)	811 (462–1306)	866 (440–1602)
**CD8(10** ^**3**^ **cells/mm** ^**3**^ **)**	468 (196–988)	505 (195–1068)	486 (201–1104)	472 (262–1167)
**CD4%**	42 (32–56)	44 (30–56)	41 (29–54)	44 (32–55)
**CD8%**	23.1(12.4–36.4)	23.5 (17.0–34.8)	24.6 (14.9–44.0)	24.3 (17.5–35.0)
**CD4:CD8 ratio**	1.8 (1.0–3.1)	1.8 (0.9–3.2)	1.6 (0.8–2.8)	1.8 (0.8–2.8)
**ALT (μ/L)**	20.5 (4.9–42.4)	17.4 (4.2–65.3)	22.4 (12.0–80.6)	18.9 (10.7–61.3)
**AST (μ/L)**	26.9 (17.0–59.2)	22.6 (12.0–43.1)	26.7 (12.5–69.3)	22.2 (13.5–48.5)
**T-Bil (μmol/L)**	13.9 (5.7–62.6)	9.7 (3.7–38.5)	13.8 (5.3–50.7)	11.5 (5.8–36.1)
**Creatinine (μmol/L)**	66.3 (49.6–103.7)	64.5 (48.0–87.6)	83.1 (54.2–137.8)	70.7 (52.4–96.8)
**Glu (mmol/L)**	3.8 (2.2–6.6)	3.8 (2.0–7.0)	3.7 (2.1–9.0)	3.8 (2.1–6.0)
**BUN (mmol/L)**	2.5 (1.7–4.1)	2.3 (1.2–4.8)	3.0 (1.8–5.3)	2.8 (1.4–4.5)

## Results

### Sample collection results

Out of 1106 participants screened for eligibility, 534 (48.3%) were males while 572 (51.7%) were females. Following screening, a total of 153 (13.8%) participants was excluded of which 125 (81.7%) were HIV-1 infected, 20 (13.1%) were pregnant and 8 (5.2%) both HIV-1 infected and pregnant. Thus, 499 (93.4%) male and 454 (79.4%) clinically healthy female participants were selected for this analysis. Of the male participants, 22.0% (110) were adolescents and 78.0% (389) were young adults while adolescents and young adults constituted 29.1% (132) and 70.9% (322) of the female participants respectively. The number of participants tested for each parameter was within the sample size (N = 120) recommended by the CLSI for the establishment of reference intervals except the male adolescent group which had 110 participants. However all gender and age groups had sample size above the number required for reference interval transference (N = 60) [10].

### Hematology and chemistry reference intervals

Tables 2 and 3 summarizes the calculated median and 95th percentile reference interval for hematological and biochemistry parameters for adolescents and young adults respectively obtained from this study. The reference intervals were generally comparable although our upper reference limit for some parameters was slightly higher than those of the established reference intervals. There were significant differences in Hb, RBC, Hct, creatinine, ALT and BUN between male and female participants in both adolescent and young adult cohorts with males having higher values but these differences were not clinically relevant (Table 4). We also observed significant differences in the hematological indices among males by age, with the young adults having a higher median as compared to adolescents in Hb (15.1 g/dL versus 14.2 g/dL), Hct (45.4% versus 42.6%), RBC (5.4X10^6^/ μL versus 5.2 X10^6^/ μL), ALT (17.4 μ/L versus 16.4 μ/L), creatinine (93 μmol/L versus 65 μmol/L) and neutrophils (2.6X10^3^/ μL versus 2.2X10^3^/ μL). Compared to the males, there was little variation in these parameters among female adolescent and adult participants except creatinine. However, females had significantly higher PLT, lymphocytes and WBC than males in both adolescent and young adult cohorts. There were significant differences in neutrophil counts between male and female adolescents, with the females having higher counts than males. There were no gender or age differences in absolute basophil, eosinophil and monocytes counts.

**Table 2:** Adolescent hematological and biochemistry reference values (median and 95^th^-percentile) comparison between locally-established reference intervals for western Kenya versus reference values established from the Kisumu Incidence cohort study in western Kenya (2007–2010).

	Local interval (Age 13–17 years) [15]		This study (Age 16–17 years)	
Parameter	Male	Female	Male	Female
**RBC (10** ^**6**^ **Cells/μl)**	4.9 (4.1–5.8)	4.7 (3.3–5.4)	5.2 (4.3–6.4)	4.9 (3.7–6.0)
**Hb (g/dL)**	13.1(10.6–15.6)	12.2 (8.1–14.2)	14.2 (11.1–16.7)	12.7 (7.5–14.8)
**HCT (%)**	38.8 (29.3–48.1)	35.6 (24.8–43.1)	42.6 (33.7–49.7)	38.0 (24.2–43.7)
**MCV (fL)**	79 (62–92)	78 (57–91)	80.9 (66.2–91.5)	79.0 (52.5–88.5)
**PLT (10** ^**3**^ **cells/μl)**	224 (103–386)	233 (134–439)	215 (112–474)	264 (126–448)
**WBC(10** ^**3**^ **cells/μl)**	5.6 (3.3–8.3)	5.2 (3.9–10.2)	5.2 (3.6–9.1)	6.0 (3.6–9.5)
**Ne (10** ^**3**^ **cells/μl)**	1.9 (0.8–5.0)	2.0 (1.1–3.1)	2.2 (0.9–6.7)	2.7 (1.3–5.8)
**Ly (10** ^**3**^ **cells/μl)**	2.2 (1.0–4.2)	2.2 (1.1–3.1)	2.2 (1.4–3.4)	2.5 (1.2–3.9)
**Mo (10** ^**3**^ **cells/μl)**	0.5 (0.2–0.7)	0.4 (0.2–0.7)	0.4 (0.2–0.8)	0.5 (0.2–1.1)
**Eo (10** ^**3**^ **cells/μl)**	0.4 (0.1–1.8)	0.4 (0.1–2.2)	0.22 (0.03–1.64)	0.24 (0.05–1.4)
**Ba (10** ^**3**^ **cells/μl)**	0.04 (0.02–0.30)	0.04 (0–0.10)	0.04 (0.02–0.09)	0.04 (0.02–0.11)
**ALT (μ/L)**	20.5 (4.9–42.4)	17.4 (4.2–65.3)	16.4 (7.8–33.9)	14.1 (5.7–32.5)
**Creatinine (μmol/L)**	66.3 (49.6–103.7)	64.5 (48.0–87.6)	65 (39–89)	51 (40–69)
**BUN (mmol/L)**	2.5 (1.7–4.1)	2.3 (1.2–4.8)	2.7 (1.2–4.5)	2.4 (1.2–4.2)

**Table 3:** Adult hematological and biochemistry reference values (median and 95^th^-percentile) comparison between locally-established reference intervals versus reference values from the Kisumu Incidence cohort study in western Kenya (2007–2010).

	Local interval (Age 18–34 years) [15]		This study (Age 18–34 years)	
Parameter	Male (n = 110)	Female (n = 132)	Male (n = 389)	Female (n = 322)
**RBC (10** ^**6**^ **Cells/μl)**	5.3 (4.3–6.5)	4.5 (3.4–5.7)	5.4 (4.6–6.6)	4.8 (4.0–5.8)
**Hb (g/dL)**	14.2 (11.4–16.9)	12.1 (8.0–14.2)	15.1 (12.6–17.2)	12.8 (9.0–14.9)
**HCT (%)**	41.7 (32.6–51.5)	35.8 (23.2–44.3)	45.4 (38.1–51.6)	38.6 (28.6–44.2)
**MCV (fL)**	80 (55–98)	79 (60–94)	84.0 (67.4–93.6)	80.4 (59.3–93.2)
**PLT (10** ^**3**^ **cells/μl)**	201 (102–307)	220 (88–439)	227 (126–356)	270 (147–454)
**WBC(10** ^**3**^ **cells/μl)**	5.3 (2.5–7.4)	5.6 (3.3–9.7)	5.6 (3.3–9.6)	5.9 (3.7–9.1)
**Ne (10** ^**3**^ **cells/μl)**	2.0 (0.8–3.9)	2.3 (1.3–3.8)	2.6 (1.3–5.2)	2.7 (1.3–5.0)
**Ly (10** ^**3**^ **cells/μl)**	2.2 (1.0–3.5)	2.2 (1.3–3.8)	2.1 (1.2–3.4)	2.3 (1.4–3.8)
**Mo (10** ^**3**^ **cells/μl)**	0.5 (0.2–0.9)	0.5 (0.3–0.8)	0.4 (0.2–0.7)	0.4 (0.2–0.8)
**Eo (10** ^**3**^ **cells/μl)**	0.5 (0.1–1.7)	0.4 (0.1–1.3)	0.23 (0.04–1.6)	0.21 (0.04–1.2)
**Ba (10** ^**3**^ **cells/μl)**	0.04 (0.01–0.19)	0.04 (0–0.20)	0.04 (0.01–0.14)	0.04 (0.02–0.09)
**ALT (μ/L)**	22.4 (12.0–80.6)	18.9 (10.7–61.3)	17.4 (8.4–54.7)	13.5 (7.2–34.1)
**Creatinine (μmol/L)**	83.1 (54.2–137.8)	70.7 (52.4–96.8)	93 (69–123)	78 (57–100)
**BUN (mmol/L)**	3.0 (1.8–5.3)	2.8 (1.4–4.5)	2.8 (1.5–5.0)	2.4 (1.2–4.1)

**Table 4:** Test of difference in hematologic and clinical chemistry parameters between gender and age-groups from the 16–34 years old cohort in Kisumu Kenya (2007–2010).

			Age 16–17 years			Age 18–34 years		
Parameter	Gender	n	median	p-value (gender)	n	median	p-value (gender)	P-value (age)
**Hemoglobin (g/dL)**	**Female**	132	12.7 (7.5–14.8)	**<*0*.*0001***	322	12.8 (9.0–14.9)	**<*0*.*0001***	*0*.*3143*
	**Male**	110	14.2 (11.1–16.7)		389	15.1 (12.6–17.2)		**<*0*.*0001***
**Hematocrit (%)**	**Female**	132	38.0 (24.2–43.7)	**<*0*.*0001***	322	38.6 (28.6–44.2)	**<*0*.*0001***	*0*.*2242*
	**Male**	110	42.6 (33.7–49.7)		389	45.4 (38.1–51.6)		**<*0*.*0001***
**WBC (x1000)**	**Female**	132	6.0 (3.6–9.5)	***0*.*0025***	322	5.9 (3.7–9.1)	***0*.*0002***	*0*.*4387*
	**Male**	110	5.2 (3.6–9.1)		389	5.6 (3.3–9.6)		*0*.*5766*
**RBC (x10** ^**12**^ **/L)**	**Female**	132	4.9 (3.7–6.0)	**<*0*.*0001***	322	4.8 (4.0–5.8)	**<*0*.*0001***	*0*.*4424*
	**Male**	110	5.2 (4.3–6.4)		389	5.4 (4.6–6.6)		**<*0*.*0001***
**Lymphocytes (x10** ^**9**^ **/L)**	**Female**	132	2.5 (1.2–3.9)	***0*.*0112***	322	2.3 (1.4–3.8)	**<*0*.*0001***	*0*.*0789*
	**Male**	110	2.2 (1.4–3.4)		389	2.1 (1.2–3.4)		***0*.*0261***
**Neutrophiles (x10** ^**9**^ **/L)**	**Female**	132	2.7 (1.3–5.8)	***0*.*0112***	322	2.7 (1.3–5.0)	*0*.*0538*	*0*.*4565*
	**Male**	110	2.2 (0.9–6.7)		389	2.6 (1.3–5.2)		***0*.*0169***
**PLT(x10** ^**9**^ **/L)**	**Female**	132	264 (126–448)	**<*0*.*0001***	322	270 (147–454)	**<*0*.*0001***	*0*.*3589*
	**Male**	110	215 (112–474)		389	227 (126–356)		*0*.*1218*
**ALT (μ/L)**	**Female**	132	14.1 (5.7–32.5)	***0*.*0030***	322	13.5 (7.2–34.1)	**<*0*.*0001***	*0*.*2750*
	**Male**	110	16.4 (7.8–33.9)		388	17.4 (8.4–54.7)		***0*.*0417***
**BUN (mmol/L)**	**Female**	132	2.4 (1.2–4.2)	***0*.*0241***	322	2.4 (1.2–4.1)	**<*0*.*0001***	*0*.*7883*
	**Male**	110	2.7 (1.2–4.5)		388	2.8 (1.5–5.0)		*0*.*0604*
**Creatinine (μmol/L)**	**Female**	132	51 (40–69)	**<*0*.*0001***	322	78 (57–100)	**<*0*.*0001***	**<*0*.*0001***
	**Male**	110	65 (39–89)		388	93 (69–123)		**<*0*.*0001***

### Comparison with locally established reference intervals, US MGH and NIH-DAIDS toxicity tables

Using the US-based MGH values, most of the KiCoS participants would have been misclassified as out of range with the highest misclassification in BUN parameter which would result in over 80% of participants excluded (Tables 5 and 6). However, using the locally established reference intervals, very few of the KiCoS participants would have been misclassified as out of range with the highest misclassification (<10%) being BUN in both adult and adolescent cohorts except males in the later (14.5%). Using the US-based MGH values, about a quarter (26.4%) of our adult female and 7.2% of adult male participants would have been misclassified as having out of range Hb levels (Table 5). In contrast, using the established reference intervals for western Kenya, only 1.2% and 1.0% of adult female and male participants would have been misclassified as having out of range Hb levels. This observation was similar for other red cell indices including Hct, MCV and RBC count with higher proportion of female participants misclassified.

**Table 5:** Out of range and frequency of adverse events in the Kisumu Adult cohort obtained from comparison with values from locally-established reference intervals, DAIDS and North American derived MGH values.

							Out of range comparison					
		This Study		Local reference [15]			MGH-USA [9]			2004 DAIDS [17]		
Parameter	Gender	95% reference interval	n	95% reference interval	n	%	95% reference interval	n	%	Cut-off	N	%
**Hemoglobin (g/dL)**	**Female**	9.0–14.9	322	8.0–14.2	4	1.2	12–16	85	26.4	>10.9	40	12.4
	**Male**	12.6–17.2	389	11.4–16.9	4	1.0	13.5–17.5	28	7.2	>10.9	3	0.8
**Hematocrit (%)**	**Female**	28.6–44.2	322	23.2–44.3	2	0.6	36–46	83	25.8			
	**Male**	38.1–51.6	389	32.6–51.5	3	0.8	41–53	37	9.5			
**MCV (%)**	**Female**	59.3–93.2	322	60–94	12	3.7	80–100	153	47.5			
	**Male**	67.4–93.6	389	55–98	3	0.8	80–100	103	26.5			
**WBC (x10** ^**9**^ **/L)**	**Female**	3.7–9.1	322	3.3–9.7	8	2.5	4.5–11.0	37	11.5	>2.5	1	0.3
	**Male**	3.3–9.6	389	2.5–7.4	0	0	4.5–11.0	82	21.1	>2.5	0	0
**RBC (x10** ^**12**^ **/L)**	**Female**	4.0–5.8	322	3.4–5.7	1	0.3	4.0–5.2	7	2.2			
	**Male**	4.6–6.6	389	4.3–6.5	2	0.5	4.2–6.3	2	0.5			
**Lymphocytes (x10** ^**9**^ **/L)**	**Female**	1.4–3.8	322	1.3–3.8	7	2.2	1.0–4.8	0	0			
	**Male**	1.2–3.4	389	1.0–3.5	4	1.0	1.0–4.8	4	1.0			
**Neutrophils (x10** ^**9**^ **/L)**	**Female**	1.3–5.0	322	1.3–3.8	10	3.1	1.8–7.7	0	0	>1.3	10	3.3
	**Male**	1.3–5.2	389	0.8–3.9	0	0	1.8–7.7	4	1.0	>1.3	13	4.0
**PLT (x10** ^**9**^ **/L)**	**Female**	147–454	322	88–439	1	0.3	150–350	12	3.7	≥125	4	1.2
	**Male**	126–356	389	102–307	4	1.0	150–350	30	7.7	≥125	8	2.1
**Eosinophils (10** ^**3**^ **cells/μl)**	**Female**	0.04–1.2	322	0–1.3	7	2.2	0–0.5	52	16.1			
	**Male**	0.04–1.6	389	0–1.7	5	1.3	0–0.5	77	19.8			
**ALT (μ/L)**	**Female**	7.2–34.1	322	0–61.3	1	0.3	0–35	7	2.2	<76.6	0	0
	**Male**	8.4–54.7	389	0–80.6	4	1.0	0–35	39	10.0	<100.8	2	0.5
**BUN (mmol/L)**	**Female**	1.2–4.1	322	1.4–4.5	23	7.1	3.6–7.1	305	94.7			
	**Male**	1.5–5.0	389	1.8–5.3	32	8.2	3.6–7.1	324	83.3			
**Creatinine (μmol/L)**	**Female**	57–100	322	0–96.8	18	5.6	0–133	0	0	<106.5	4	1.2
	**Male**	69–123	389	0–137.8	2	0.5	0–133	2	0.5	<151.6	1	0.3

**Table 6:** Out of range and frequency of adverse events in the Kisumu Adolescent cohort obtained from comparison with values from locally-established reference intervals and DAIDS values.

							Out of range comparison					
		This Study		Local reference [15]			MGH-USA [9]			2004 DAIDS [17]		
Parameter	Gender	95% reference interval	n	95% reference interval	n	%	95% reference interval	n	%	Cut-off	N	%
**Hemoglobin (g/dL)**	**Female**	7.5–14.8	132	8.1–14.2	7	5.3	12–16	37	28.0	>10.9	18	13.6
	**Male**	11.1–16.7	110	10.6–15.6	1	0.9	13.5–17.5	36	32.7	>10.9	2	1.8
**Hematocrit (%)**	**Female**	24.2–43.7	132	24.8–43.1	2	1.5	36–46	30	22.7			
	**Male**	33.7–49.7	110	29.3–48.1	2	1.8	41–53	41	37.3			
**MCV (%)**	**Female**	59.3–93.2	132	57–91	6	4.5	80–100	76	57.6			
	**Male**	67.4–93.6	110	62–92	1	0.9	80–100	48	43.6			
**WBC (x10^9^/L)**	**Female**	3.6–9.5	132	3.9–10.2	3	2.3	4.5–11.0	12	9.1	>2.5	3	2.3
	**Male**	3.6–9.1	110	3.3–8.3	0	0	4.5–11.0	27	24.5	>2.5	0	0
**RBC (x10^12^/L)**	**Female**	3.7–6.0	132	3.3–5.4	1	0.8	4.0–5.2	4	3.0			
	**Male**	4.3–6.4	110	4.1–5.8	2	1.8	4.2–6.3	2	1.8			
**Lymphocytes (x10** ^**9**^ **/L)**	**Female**	1.2–3.9	132	1.1–3.1	4	3.0	1.0–4.8	2	1.5			
	**Male**	1.4–3.4	110	1.0–4.2	0	0	1.0–4.8	0	0			
**Neutrophiles (x10** ^**9**^ **/L)**	**Female**	1.3–5.8	132	1.1–3.1	3	2.3	1.8–7.7	20	15.2	>1.3	3	2.3
	**Male**	0.9–6.7	110	0.8–5.0	0	0	1.8–7.7	27	24.5	>1.3	9	8.2
**PLT (x10** ^**9**^ **/L)**	**Female**	126–448	132	134–439	2	1.5	150–350	6	4.5	≥125	3	2.3
	**Male**	112–474	110	103–386	1	0.9	150–350	14	12.7	≥125	8	7.3
**Eosinophils (10** ^**3**^ **cells/μl)**	**Female**	0.04–1.2	132	0–2.2	1	0.8	0–0.5	24	18.2			
	**Male**	0.04–1.6	110	0–1.8	0	0	0–0.5	22	20.0			
**ALT (μ/L)**	**Female**	5.7–32.5	132	0–65.3	1	0.8	0–35	3	2.3	<76.6	0	0
	**Male**	7.8–33.9	110	0–42.4	1	0.9	0–35	2	1.8	<100.8	0	0
**BUN (mmol/L)**	**Female**	1.2–4.2	132	1.2–4.8	4	3.0	3.6–7.1	116	87.9			
	**Male**	1.2–4.5	110	1.7–4.1	16	14.5	3.6–7.1	96	87.3			
**Creatinine (μmol/L)**	**Female**	40–69	132	0–87.6	0	0	0–133	0	0	<106.5	0	0
	**Male**	39–89	110	0–103.7	0	0	0–133	0	0	<151.6	0	0

Using the 2004 NIH DAIDS toxicity grading to select participants eligible for the study (Tables 5 and 6), 12.8% (n = 58) of female participants and 1.0% (n = 5) of male participants would have been classified as having an abnormal Hb level. However, only 2.4% (n = 11) of the female participants and 1.0% (n = 5) of male participants would have been classified as having out of range values using the locally established reference intervals. Similarly, 3.4% (n = 17) male and 1.5% (n = 7) female participants would have been classified as having an abnormal platelet count using the 2004 NIH DAIDS toxicity grading while only 1.0% (n = 5) male and 0.7% (n = 3) female participants would have been classified as out of range using the established reference interval for western Kenya.

## Discussion

With increasing clinical trials in Africa in an effort to combat tropical diseases [24], a need arises to consider the health status of the likely participants in such studies [12]. In this regard, several African studies have generated reference intervals for use in the respective regions [13, 15, 24, 25]. While it is important to develop locally derived reference intervals that ensure proper assessment of volunteers in clinical trials, monitoring of laboratory-based adverse events and prevention of unnecessary exclusion, it is important to evaluate their use within the local population. To our knowledge, this is one of the first evaluations of established reference intervals reported in sub-Saharan Africa. In this study, we evaluate the use of hematological and biochemistry reference intervals established for western Kenya using specimen drawn from participants in a HIV incidence cohort study in Kisumu. Our values were comparable to those of the established reference intervals for most parameters although our median values were slightly higher for most hematological parameters. This may be so given that the samples for this evaluation were drawn from an urban population that may have had access to better healthcare, clean water and nutrition than the rural population from where the established reference intervals for western Kenya [15] were obtained. Moreover, using the US MGH reference interval, the overall out of range MCV values constituted 54.0% [15] of the study population in rural western Kenya but only 27.6% in this study. Low MCV is an indirect marker of iron deficiency [26]. This is further corroborated by the low eosinophil counts observed in this study. Our eosinophil counts are comparable to those obtained from an urban population of blood donors in Uganda [18] in contrast to higher counts in a similar study in a rural population in the same country [27]. Our values for ALT and creatinine were also lower than those of Zeh et al [15]. Similarly, a study in Cameroon designed to establish reference intervals for biochemical parameters reported statistical differences in biochemistry reference parameters between participants from urban and rural geographic regions [28]. While this might not necessitate the need to establish separate intervals, consideration should be made when applying such intervals within specific populations.

Using the US-based MGH reference intervals to hypothetically select participants in a trial based on Hb, WBC counts, neutrophil counts, eosinophil counts and platelets, 51.5% (n = 491) of the total participants would have been excluded from participating in the study (Table 7). However, using the established reference intervals for western Kenya, only 6.7% (n = 64) of participants would have been excluded from participating in the study. Including BUN in the selection criteria would result in exclusion of over 80% of participants. This was similar in other African studies [7, 13, 15, 27] suggesting that this may result from a common environmental or genetic factor [12, 13]. Thus use of locally established reference intervals would reduce the overall screening to enrollment ratio in this case. This reduces the overall cost of screening and theoretically would reduce the time period for screening by reaching the study target within a shorter time period. Eller et al. have documented similar findings in a study of healthy adult Ugandan blood donors [18]. It is not surprising to see that the adolescent cohort resulted in the most out of range values hence yielded the least enrolled participants using the western-derived reference intervals and toxicity tables. Thus, partitioning of male adolescents needs to be considered in future trials.

**Table 7:** Hypothetical enrollment using local reference intervals compared to US-derived reference intervals and the DAIDS toxicity tables.

			Number (%) Enrolled		
Age category	Sex	No of participants	Local intervals [15]	US MGH [9]	*DAIDS [17]
**Adolescents**	**Male**	110	106 (96.4%)	27 (24.5%)	70 (63.6%)
	**Female**	132	113 (85.6%)	67 (50.8%)	81 (61.4%)
**Adults**	**Male**	389	374 (96.1%)	196 (50.4%)	289 (74.3%)
	**Female**	322	296 (92.0%)	172 (53.4%)	218 (67.7%)
	**Total**	953	889 (93.3%)	462 (48.5%)	658 (69.0%)

* eosinophil count grading using US derived values for adults.

Similarly, using the 2004 NIH US DAIDS toxicity grading for screening, 31.0% (n = 295) of participants would have been excluded from the study. Although the table has been revised for some parameters including neutropenia [29], a large proportion of our study participants would still have been excluded based on Hb levels. Moreover, the toxicity table does not take into account the significant difference in red blood cell parameters between males and females, thus, a majority of those excluded would constitute female participants.

The CLSI guidelines recommend the collection of specimen from healthy volunteers for use in validating reference intervals. Thus, a limitation of this study was the failure to screen for possible asymptomatic parasitic infections like malaria and helminthes which are endemic within the study region. However, our Hb values were much higher than those from a study within the same region that screened out malaria infected participants [13]. Moreover, our eosinophil counts were much lower than the two studies within the region which screened participants from a rural population [13, 15]. A second limitation may have been that this was a self-selected population of participants willing to participate in a cohort study. However, this represents a similar population that would be willing to participate in a clinical trial thus provides a good sample to evaluate the use of the locally established reference intervals. Moreover, the HIV prevalence of the study population (12.0%) is comparable to the prevalence within the general population [30].

Given that the number of clinical trials and persons receiving clinical services is expected to increase substantially in sub-Saharan Africa, there is a need for the establishment and evaluation of locally derived clinical laboratory reference values to ensure appropriate general health assessment, treatment monitoring, and efficient implementation of clinical trials. Even more important is the need for the establishment of toxicity grading tables for application in clinical care among Africans based on the documented differences between laboratory reference intervals from African and Caucasian populations. This study confirms that the hematological and biochemistry reference intervals established by Zeh et al. are valid for use in participant recruitment in western Kenya.
